# Kyphoscoliosis with Klippel-Trenaunay syndrome: a case report and literature review

**DOI:** 10.1186/s12891-018-2393-z

**Published:** 2019-01-05

**Authors:** Jiliang Zhai, Min-Er Zhong, Jianxiong Shen, Haining Tan, Zheng Li

**Affiliations:** 1 Department of Orthopaedic Surgery, Peking Union Medical College Hospital, Chinese Academy of Medical Science and Peking Union Medical College, Beijing, 100730 China; 2 Department of General Surgery, Peking Union Medical College Hospital, Chinese Academy of Medical Sciences and Peking Union Medical College, Beijing, 100730 China

**Keywords:** Klippel-Trenaunay syndrome, Kyphoscoliosis, Scoliosis, Venous varicosities, Capillary malformations, Limb hypertrophy

## Abstract

**Background:**

Klippel-Trenaunay syndrome (KTS) is a rare congenital syndrome characterized by the triad of venous varicosities, capillary malformations and limb hypertrophy. However, KTS may rarely occur in combination with kyphoscoliosis.

**Case presentation:**

We presented an 18-year-old female with KTS and kyphoscoliosis. Hypertrophy of bone and soft tissue affected her left face, trunk and lower limb. Moreover, the patient is associated with subacute thyroiditis, vitamin D deficiency and iron deficiency anemia, high level of D-dimer, swollen tonsil, kyphoscoliosis and Chiari-I-malformation without syringomyelia. A posterior correction and spinal fusion from T10 to L5 levels were performed for this patient. The lumbar curve was corrected from 105° to 60° and the kyphosis improved from 58° to 26°. The distance of trunk shift decreased from 10 cm to 1.4 cm. There were no thrombotic events occurred. At the 8th month follow-up, there was no significantly change of the curve in the coronal and sagittal radiographs. During the 31-month follow-up, the patient did not experience any discomfort. And her general appearance did not have any change until the last follow-up. However, she refused to take radiograph for worrying about radiation.

**Conclusions:**

KTS is a rare disease with classic clinical triad. However, it can also have other different features, including kyphoscoliosis, elevated D-Dimer, vitamin D deficiency and iron-deficiency anemia. These issues should be taken into consideration when planning treatment for kyphoscoliosis in KTS patients.

## Background

Klippel-Trenaunay Syndrome (KTS), a syndrome of capillary-lymphatic-venous malformation associated with soft tissue and skeletal hypertrophy, is a rare congenital disorder. It has a very low incidence of about 1:100,000. It has no predilection for gender, race, or geographical area and occurs sporadically [1]. In 1900, Maurice Klippel and Paul Trenaunay described two patients with asymmetric soft tissue and bone hypertrophy [2]. The term KTS has since been used to describe congenital, complex malformation identified by the classic clinical triad of cutaneous capillary malformations (port wine stain), soft tissue and bone hypertrophy, and venous varicosities. However, KTS may rarely occur in combination with kyphoscoliosis. In current study, we presented an 18-year-old female patient with KTS, who also suffered from kyphoscoliosis.

## Case presentation

**Fig. 1:**
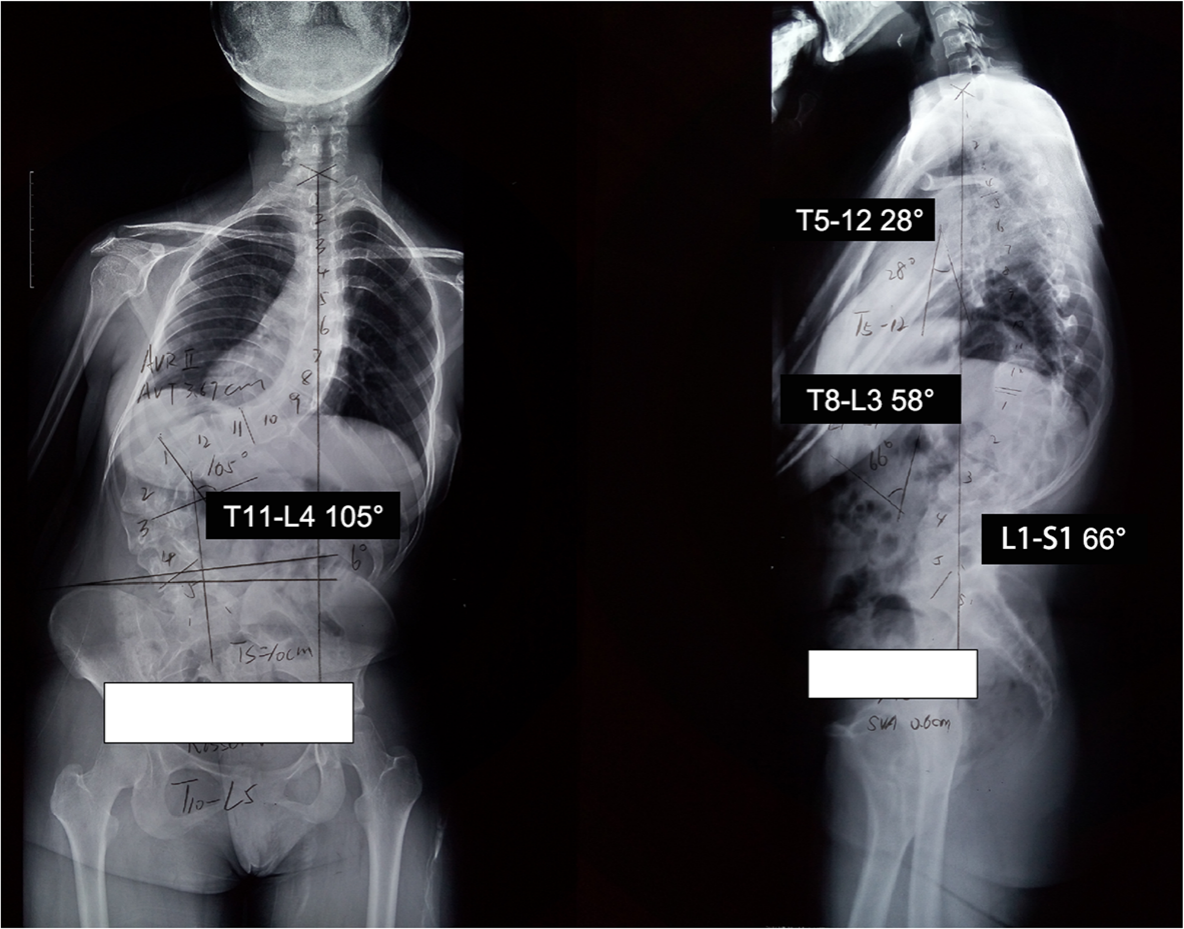
Standing anteroposterior and lateral radiographs

An 18-year-old female patient was admitted to our hospital with complaint of kyphoscoliosis after birth. Her radiographs with the spine demonstrated that the Cobb angle of lumbar scoliosis was 105° (Thoracic 11 to Lumbar 4) and the distance of trunk shift was 10 cm. The kyphosis angle from T8 to L3 was 58° (Fig. 1).

At birth, the patient was noted to have hemihypertrophy and hemangioma on her face and back. Abnormal asymmetric growth became apparent along with her age. She underwent laser therapy for her facial hemangioma at the age of 4. Three years ago, venous varicosities appeared on both lower extremities. Klippel-Trenaunay Syndrome (KTS) was diagnosed for her. In addition, the patient had a history of hypoxic-ischemic encephalopathy (HIE) at birth. However, the Apgar score was unclear. Her mother took some medicine for cold at her 8th week of pregnancy.

There was no family history of KTS.

**Fig. 2:**
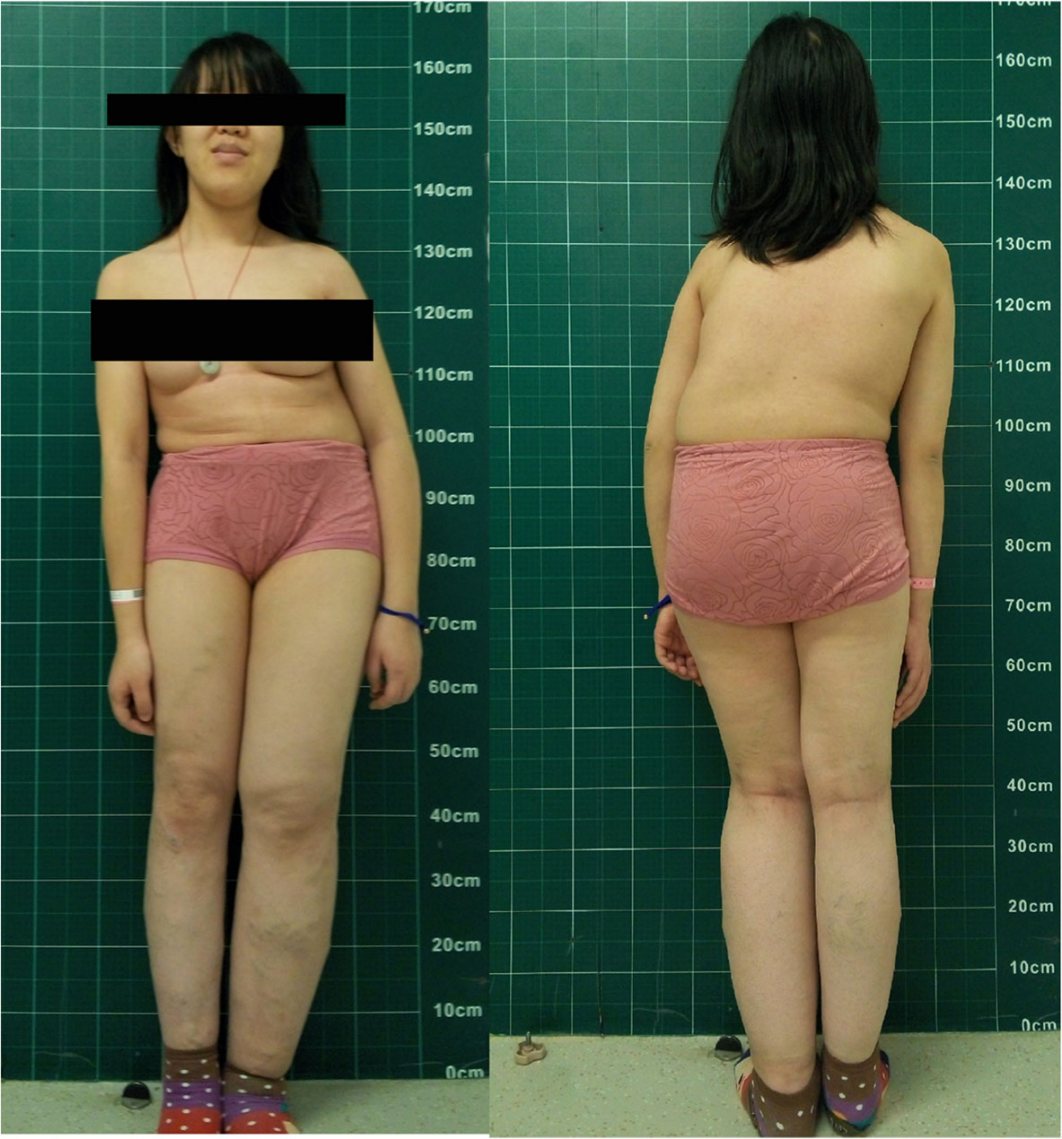
Standing anteroposterior and lateral view show hemihypertrophy of the left face, trunk, upper and lower extremities

**Fig. 3:**
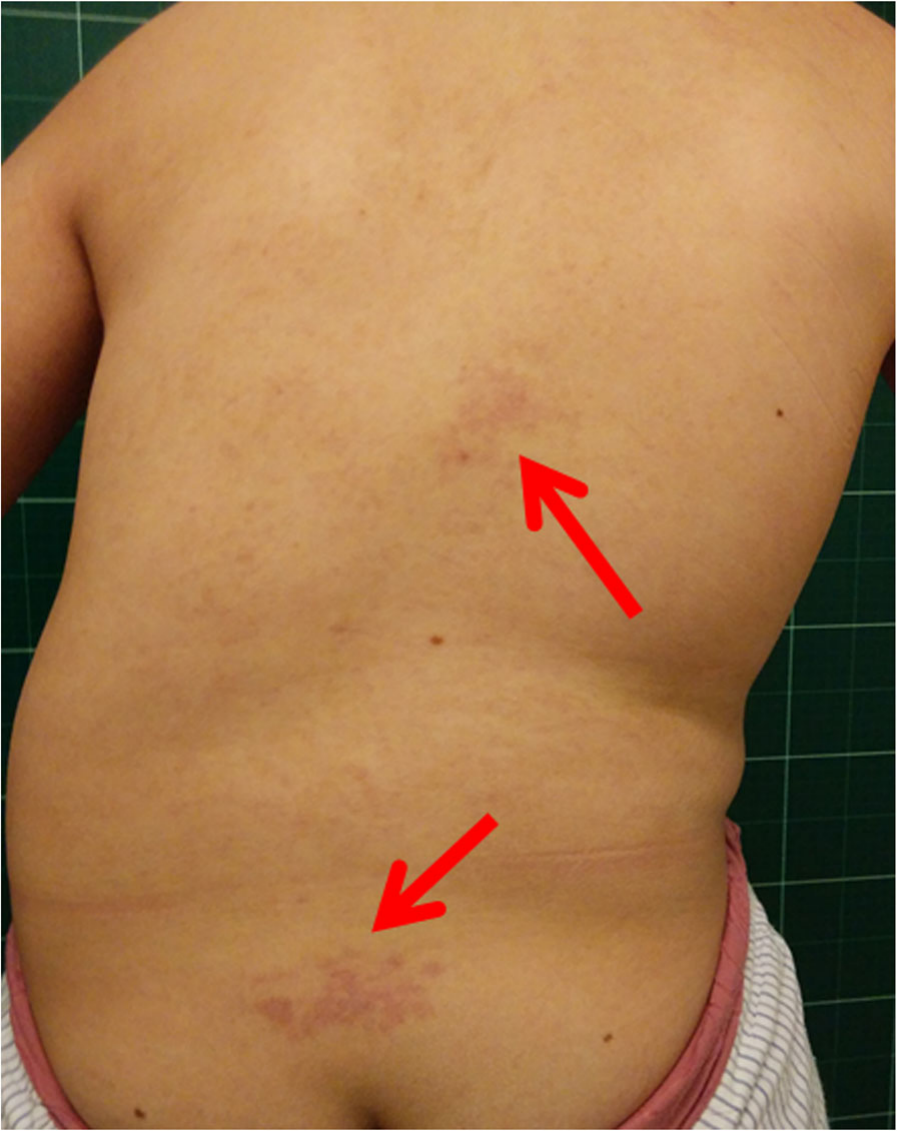
Port-wine stain on the back (red arrows)

**Fig. 4:**
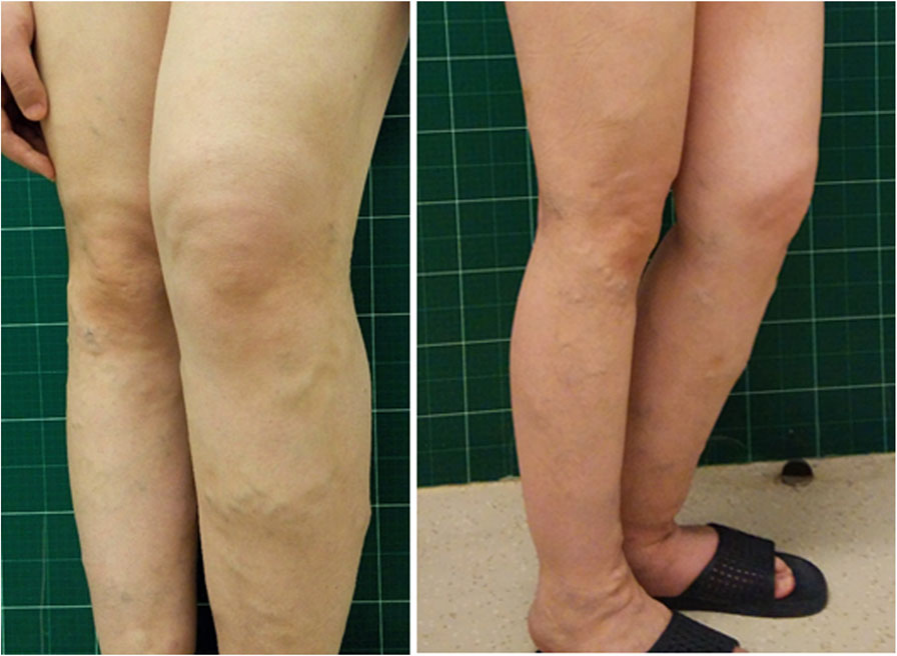
Venous varicosities of both the lower limbs

Physical examination showed hemihypertrophy of the left face, trunk, lower limb (Fig. 2). There was a port-wine stain on her back (Fig. 3) and venous varicosities on both lower limbs (Fig. 4). Her left tonsil was swollen in 3 degrees. Her left leg was 2 cm longer than the right side. Obvious claudication was noted when she walked. Neurological examination was intact.

Positive laboratory examination results included an increased D-Dimer level of 2.02 mg/L (0~0.55, FEU), decreased hemoglobin level of 105 g/L (110–150 g/L), decreased 1,25(OH)2D_3_ level of 6.58 pg/mL (19.6~54.3 pg/mL), decreased Fe level of 34.7μg/dL (50~170 μg/dL), and decreased ferritin (Fer) level of 8 ng/mL (14~307 ng/mL). Thyroid function test showed increased TSH of 6.669 μIU/mL, A-Tg of 189.20 IU/mL, and A-TPO of 297.50 IU/mL. The fecal occult blood test was negative.

A computed tomographic (CT) scan of spine revealed no vertebral body deformities. Doppler ultrasound scan found no significant arteriovenous shunting. A magnetic resonance imaging of the spine showed Chiari-I-malformation without syringomyelia.

**Fig. 5:**
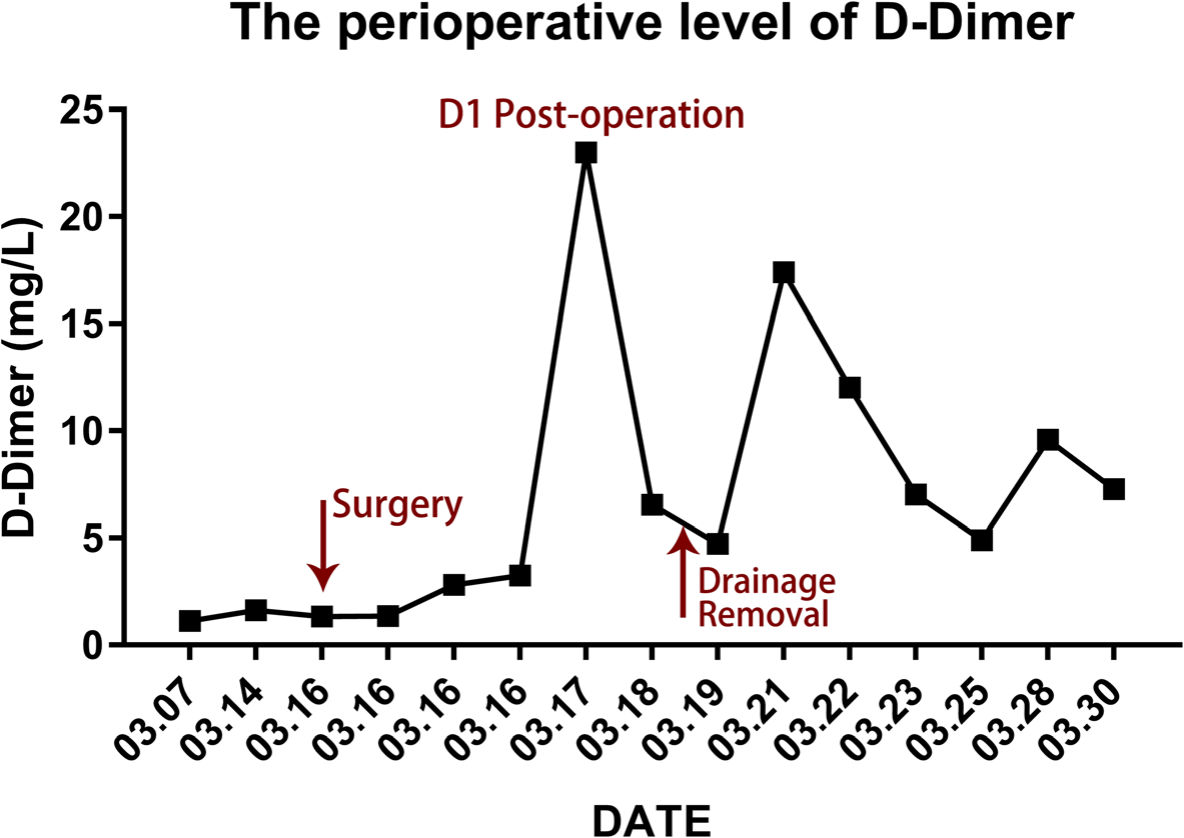
The perioperative level of D-Dimer

**Fig. 6:**
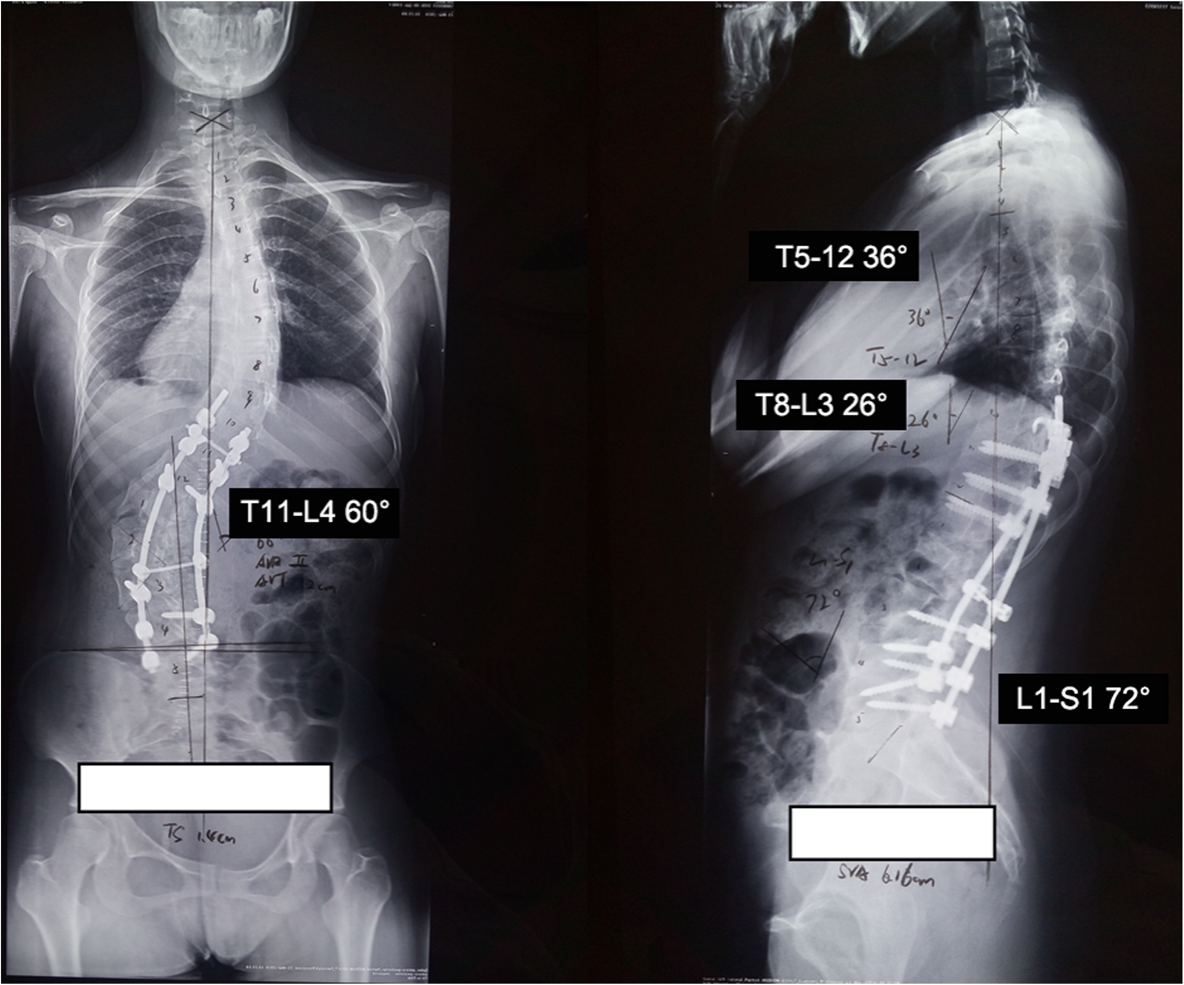
Post-operative standing anteroposterior and lateral radiographs

**Fig. 7:**
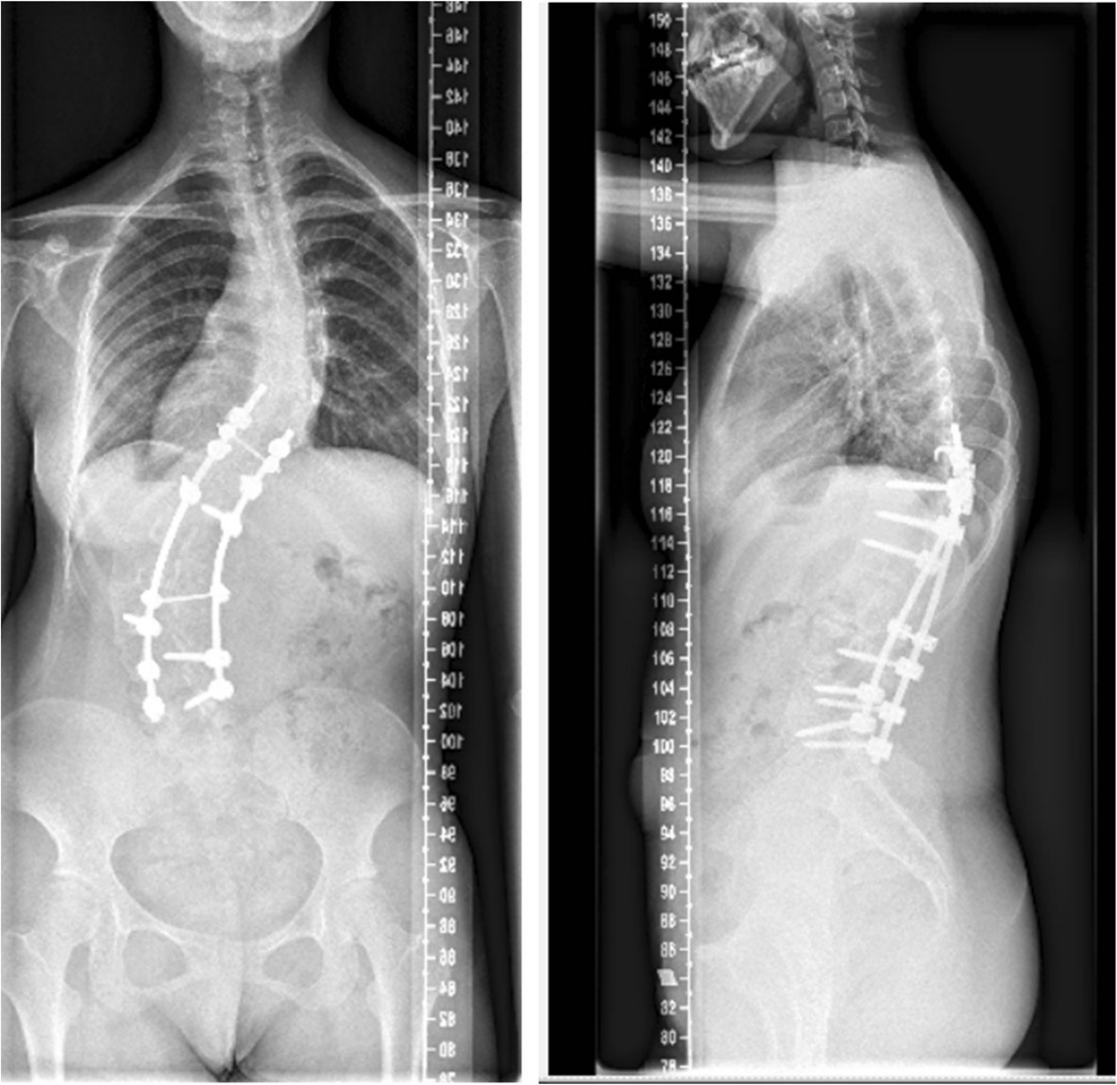
Standing anteroposterior and lateral radiographs of 3 months after operation

**Fig. 8:**
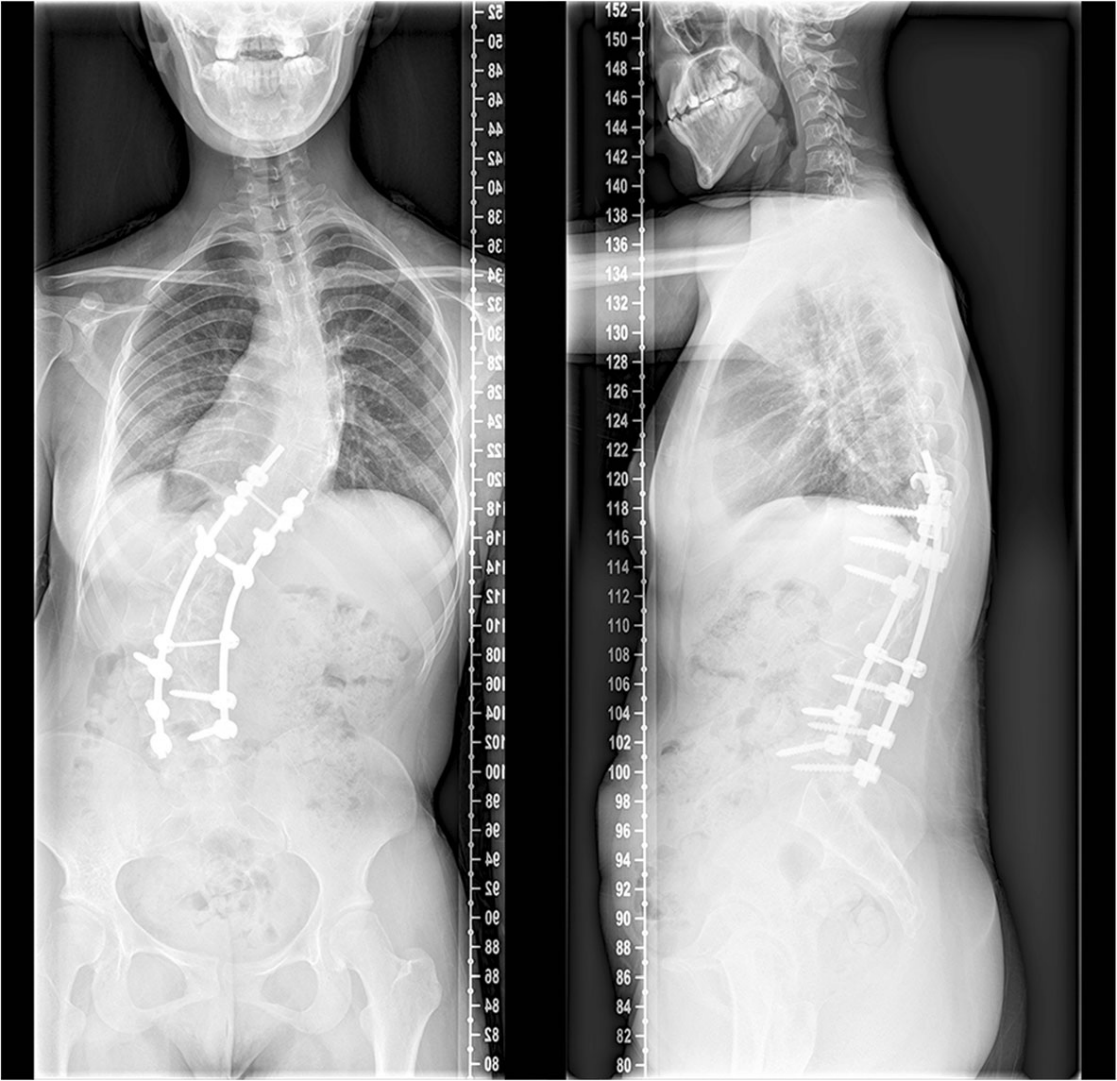
Standing anteroposterior and lateral radiographs of 8 months after operation

We had a consultation with endocrinologist and hematologist. However, the relation between kyphoscoliosis and other comorbidities could not be determined. Vitamin D deficiency, like other comorbidities, might be due to innutrition. Oral ferrous succinate and cholecalciferol cholesterol emulsion were administrated preoperatively. As a result, her Hemoglobin, Fe, and Fer regained normal value before surgery. However, her 1,25(OH)2D_3_ level was 5.21 pg/mL, which was still lower than the normal value. Subcutaneous injection of low molecular weight heparin was conducted preoperatively and maintained two weeks postoperatively. The dynamic change of D-Dimer level was shown in Fig. 5. Finally, posterior scoliosis correction and spinal fusion from T10 to L5 levels were performed. During surgery, we found that the scoliosis was very rigid and blood oozing from the wound surface was obvious. Bone quality was similar to other adolescent patients during pedicle screw implantation, although the patient had Vitamin D deficiency. Left pedicle of T10 poorly developed and we failed to place left pedicle screw of T10. The total operation time was about 5 hours and the amount of blood loss was 1300 mL. The motor evoked potential signal of the spinal cord was normal during the operation. Postoperative plain X-ray film demonstrated the Cobb angle of lumbar curve corrected from 105° to 60° (correction rate 43%) and the distance of trunk shift decreased from 10 cm to 1.4 cm (Fig. 6). The kyphosis angle decreased from 58° to 26°. No thrombotic events or other complications occurred during perioperative period. At the 3rd month follow-up, there was no change of the curve in the coronal and sagittal planes (Fig. 7). At the 8th month follow-up, the Cobb angle in the coronal and sagittal planes was 54° and 34°, respectively. The trunk shift was 1.5 cm in the coronal plane, which was not significantly different from that of postoperative (Fig. 8). During the 31-month follow-up, the patient did not experience any discomfort. And her general appearance did not have any change until the last follow-up. However, she refused to take radiograph for worrying about radiation.

## Discussion and conclusion

Klippel-Trenaunay Syndrome (KTS) is known as a complex congenital disease occurs sporadically. It is characterized by typical triad (capillary malformations, venous varicosities, limb hypertrophy) with or without lymphangiopathy. KTS is a rare disorder of obscure etiology and pathogenesis. In most cases, KTS triad limited to one extremity. Nevertheless, Jacob et al. had reported more than 70 patients had involvement of bilateral extremity [3]. The site of involvement could be various from one to another while the leg is the most commonly affected site. Patients can be diagnosed with KTS with one or more of the triad features [4]. Jacob et al. reviewed the clinical characteristics in 252 KTS patients at Mayo Clinic, and found that 63% patients had all three typical features and the rest 37% were affected with two of the three features [3]. Capillary malformations, usually referred to as port wine stains, were found in most patients with KTS (98%) and often the first abnormality to be recognized. Varicosities or venous malformations and limb hypertrophy were found in 72 and 67% of the patients, respectively. Most previous studies reached a similar result with Jacob and his colleagues’. While most studies have found that nearly all of KTS patients have port wine stains, Servelle [5] claimed that cutaneous vascular anomaly was absent in 68% of cases in a series of 614 KTS patients. In Servelle’s report, varicose veins appeared in more than 76% of patients. It is remarkable that varicose veins or venous malformations in KTS may not be apparent immediately after birth and become more evident with increasing age [6]. Most KTS patients were found with hypertrophy of bone and soft tissue [3, 5]. Although any part of the body can be involved, the most commonly affected sites are the lower extremities [7]. Limb findings varied from finger and toe deformities such as acrodactyly, polydactyly, syndactyly, metatarsal and phalangeal agenesis to osteolysis, congenital dislocation of the hip and peripheral neuropathy [2, 8]. As for vascular malformations, almost all organs of the body can be affected, including cardiovascular system, gastrointestinal tract, liver, spleen, and genitourinary tract. [9, 10]

The patient in this study has all the features of typical KTS triad. Hypertrophy of bone and soft tissue affected her left lower extremity, face and trunk. Her facial and dorsal hemangioma is the manifestation of the cutaneous capillary malformation. Varicose veins are obvious on her lower extremities. Moreover, the patient is associated with subacute thyroiditis, vitamin D deficiency and iron deficiency anemia, high level of D-dimer, swollen tonsil, kyphoscoliosis and Chiari-I-malformation without syringomyelia.

KTS was reported to associate with other congenital malformations, such as congenital dislocation of the hip (CDH) [8] and spinal arteriovenous malformation [11]. However, KTS may rarely occur in combination with kyphoscoliosis. Arai Y et al. [12] reported a case of myelopathy due to scoliosis in Klippel-Trenaunay-Weber syndrome and this patient underwent decompression of hypertrophic bone. However, they did not correct the scoliosis. The exact cause and mechanism of scoliosis in the patient in this study is unknown. It may secondary to limb length discrepancy (about 2 cm), striking pelvic obliquity and long-term claudication. Another hypothesis is that her scoliosis is closely related to her Chiari-I-malformation without syringomyelia. As we know, scoliosis is commonly associated with Chiari-I-malformation with the concomitant rate from 15 to 50% [13]. Moreover, 10.5%~ 27.3% of Chiari-I-malformation patients with scoliosis do not have concurrent syringomyelia [14], just like in this case. The scoliosis may also be the result of KTS due to asymmetric growth of the spinal column.

It is uncommon in an adolescent to have vitamin D deficiency and iron-deficiency anemia. Her serum ferritin level is extremely low. Repeated fecal occult blood test and Sudan III staining showed negative test result. Besides, her iron deficiency is worse than anemia. It seems that she is at a condition of innutrition. Her elevated D-Dimer may be explained by extensive venous malformations. The association between extensive venous malformations and hypercoagulability was established a long time ago. Previous studies found that patients with large venous malformations has chronic low-grade consumptive coagulopathy [15]. Mazoyer [16] proposed that the coagulopathy among patients with venous malformations was a result of localized intravascular coagulation.

There is currently no cure for KTS, but the symptoms associated with it can be improved with treatment. For example, the intensity of capillary malformation can be decreased by laser therapy and sclerotherapy. Venous varicosities can be ameliorated by physiotherapy and compression [17]. Since the severity of symptoms of KTS varies from person to person, treatment regimens should be tailored to the specific situation of the patient. In the present study, the patient needs surgery for severe scoliosis and trunk shift, and it is best to fused from T2 to L5 to correct the scoliosis. However, long segmental correction surgery will take longer time and cause more blood loss or even disseminated intravascular coagulation (DIC), especially for the patient with coagulopathy. Ogura Y et al. [18] reported a special case of scoliosis with huge subcutaneous cavernous hemangioma who underwent posterior correction and fusion surgery. Unfortunately, massive hemorrhage occurred and the intraoperative blood loss was up to 2800 mL. Consequently, the patient suffered from perioperative complications including hypovolemic shock, DIC and sensory and conduction aphasia due to cerebral hypoxia. Considering the high risk of massive hemorrhage and secondary complications, we would like to perform short segmental fusion to correct trunk shift, instead of longer segmental scoliosis correction. Although short segment surgery was performed, the operation time was about 5 h and blood loss was about 1300 mL.

KTS is a rare disease with classic clinical triad of cutaneous capillary malformations, soft tissue and bone hypertrophy, and venous varicosities. The patient in this study has different features from the literatures including elevated D-Dimer, vitamin D deficiency and iron-deficiency anemia. Posterior correction and spinal fusion from T10 to L5 levels were performed and the lumbar curve corrected from 105° to 60° (correction rate 43%). This is the first in literature to highlight the management of kyphoscoliosis in KTS.

## Abbreviations

CDH: congenital dislocation of the hip
CT: computed tomographic
DIC: disseminated intravascular coagulation
HIE: hypoxic-ischemic encephalopathy
KTS: Klippel-Trenaunay Syndrome
